# Deucravacitinib in Japanese patients with plaque, generalized pustular, or erythrodermic psoriasis: Patient‐reported outcomes in the POETYK PSO‐4 study

**DOI:** 10.1111/1346-8138.17570

**Published:** 2024-12-06

**Authors:** Yayoi Tada, April W. Armstrong, Shinichi Imafuku, Yukari Okubo, Akimichi Morita, Yichen Zhong, Joe Zhuo, Brandon Becker, Andrew Napoli, Subhashis Banerjee, Mamitaro Ohtsuki

**Affiliations:** ^1^ Department of Dermatology Teikyo University School of Medicine Tokyo Japan; ^2^ Department of Dermatology, Keck School of Medicine University of Southern California Los Angeles California USA; ^3^ Division of Dermatology University of California Los Angeles Los Angeles California USA; ^4^ Department of Dermatology Fukuoka University Hospital Faculty of Medicine Fukuoka Japan; ^5^ Department of Dermatology Tokyo Medical University Tokyo Japan; ^6^ Department of Geriatrics and Environmental Dermatology Nagoya City University Graduate School of Medical Science Nagoya Japan; ^7^ Bristol Myers Squibb Princeton New Jersey USA; ^8^ Jichi Medical University Tochigi Japan

**Keywords:** clinical trial, patient‐reported outcome measures, psoriasis, quality of life, treatment outcome

## Abstract

Deucravacitinib, an oral, selective, allosteric tyrosine kinase 2 inhibitor, is approved in Japan for adult patients with plaque, generalized pustular, or erythrodermic psoriasis. POETYK PSO‐4 (NCT03924427), an open‐label, single‐arm, phase 3 trial, showed that deucravacitinib was effective and well tolerated in Japanese patients with plaque (*n* = 63), generalized pustular (*n* = 3), or erythrodermic (*n* = 8) psoriasis. Additional end points in POETYK PSO‐4 included change measured by the patient‐reported outcome measures Psoriasis Symptoms and Signs Diary and Dermatology Life Quality Index. Mean changes from baseline in score on each measure and the response rate for achieving Dermatology Life Quality Index scores of 0 or 1 were assessed in each patient group over 52 weeks. All assessments were reported as observed, without imputation, in the as‐treated population. Each group reported week 16 score improvements from baseline for both patient‐reported outcome measures that were maintained or numerically improved at week 52. At week 52, approximately two‐thirds of patients in each group achieved a Dermatology Life Quality Index score of 0 or 1, indicating no impact of disease on quality of life. These results demonstrate improvements in psoriasis symptoms and signs and in quality of life with deucravacitinib treatment in Japanese patients with plaque, generalized pustular, or erythrodermic psoriasis. The small number of patients with generalized pustular or erythrodermic psoriasis limits the generalized interpretability of the findings in these groups.

## 1 INTRODUCTION

Psoriasis is a chronic, immune‐mediated inflammatory disease with an estimated prevalence of 0.3% in Japan.^1^ Plaque psoriasis (PP) accounts for up to 97.4% of psoriasis cases in Japan, while the generalized pustular psoriasis (GPP) and erythrodermic psoriasis (EP) subtypes represent 1.1% and 0.4% of cases, respectively.^1^

Deucravacitinib, an oral, selective, allosteric tyrosine kinase 2 (TYK2) inhibitor, is approved in Japan and other countries for adults with moderate to severe PP^2^, ^3^, ^4^ and in Japan for patients with GPP and EP who have an inadequate response to conventional systemic treatments.^2^ In the global phase 3 POETYK PSO‐1 (NCT03624127)^5^ and POETYK PSO‐2 (NCT03611751)^6^ trials, deucravacitinib was significantly more effective than placebo or apremilast at 16 weeks and was well tolerated over 52 weeks in patients with moderate to severe PP.^5^, ^6^ In addition, POETYK PSO‐4 (NCT03924427),^7^ an open‐label, single‐arm, phase 3 trial assessing the efficacy and safety of deucravacitinib in Japanese patients with moderate to severe PP, GPP, or EP, found that deucravacitinib was effective and well tolerated over 52 weeks in patients with moderate to severe PP and in the limited number of enrolled patients with GPP or EP.^7^ However, as psoriasis is associated with a high symptom burden that may profoundly affect patients' physical, emotional, and social quality of life (QoL),^8^ demonstration of improvements in patient‐reported outcomes (PROs) is crucial. Here, we describe improvements with deucravacitinib in the PRO measures Psoriasis Symptoms and Signs Diary (PSSD) and Dermatology Life Quality Index (DLQI) in POETYK PSO‐4.

## 2 METHODS

### 2.1 Study design and population

The phase 3, open‐label, single‐arm POETYK PSO‐4 trial was conducted at 25 sites in Japan.^7^ Japanese patients aged ≥18 years were included. Eligible patients received open‐label treatment with deucravacitinib 6 mg once daily for 52 weeks.

### 2.2 Assessments

The PSSD, a validated instrument in psoriasis,^9^ assesses the severity of five skin symptoms (itch, pain, stinging, burning, and tightness) and six patient‐reported skin signs (dryness, cracking, scaling, shedding or flaking, redness, and bleeding) over the previous 24 h. Each item uses a numeric rating scale ranging from 0 (absent) to 10 (worst imaginable). Symptom and sign summary scores are generated by averaging item scores in each domain and multiplying by 10. Patients completed the PSSD daily, with weekly scores representing the average of daily scores over 7 days. Mean changes from baseline to 52 weeks in PSSD scores are reported for the three patient groups (PP, GPP, or EP). Papp et al have established that a ≥2‐point improvement from baseline score on individual PSSD items represents meaningful within‐patient change^10^; response rates for meaningful change on individual PSSD items are reported for the PP group.

The DLQI comprises questions about the QoL impact of 10 items across six domains; each question uses a 4‐point Likert‐type scale ranging from 0 (not at all) to 3 (very much). Score decreases of ≥4 points from baseline represent meaningful within‐patient change.^11^ Mean DLQI score change from baseline, response rates for meaningful change, and response rates for DLQI scores of 0 or 1 (DLQI 0/1) in patients with baseline scores of ≥2 are reported over 52 weeks for all three psoriasis groups.

All assessments are reported as observed, without imputation, for the as‐treated population (all enrolled patients who received one or more treatment dose).

## 3 RESULTS

### 3.1 Study population

The as‐treated population included 74 patients (PP, *n* = 63; GPP, *n* = 3; EP, *n* = 8).^7^ The mean (standard deviation [SD]) age among the populations was 48.6 years (11.7 years) and most patients were male (*n* = 57; 77.0%). Baseline demographics and clinical characteristics are presented in Table 1.

**TABLE 1:** Demographics and baseline disease characteristics.

Parameter	Plaque psoriasis (*n* = 63)	Generalized pustular psoriasis (*n* = 3)	Erythrodermic psoriasis (*n* = 8)
Age, mean (SD), years	49.1 (12.1)	43.3 (17.6)	46.5 (5.4)
Female, *n* (%)	15 (23.8)	2 (66.7)	0 (0.0)
Weight, mean (SD), kg	69.5 (14.2)	72.4 (7.0)	78.8 (16.0)
sPGA score,^a^ *n* (%)			
3	56 (88.9)	2 (66.7)	7 (87.5)
4	7 (11.1)	0 (0.0)	1 (12.5)
PASI score, mean (SD)	21.1 (9.2)	20.8 (12.3)	44.9 (10.6)
BSA involvement, mean (SD), %	30.3 (18.6)	37.0 (15.4)	86.8 (6.2)
PSSD score, mean (SD)			
Symptom	39.8 (23.6)	46.6 (4.0)	35.7 (26.0)
Sign	48.0 (19.5)	57.3 (20.3)	47.1 (19.0)
DLQI score, mean (SD)	9.1 (4.5)	9.7 (6.4)	7.5 (5.6)

*Note*: Adapted with permission from Imafuku S, et al.^7^

Abbreviations: BSA, body surface area; DLQI, Dermatology Life Quality Index; GPP, generalized pustular psoriasis; PASI, Psoriasis Area and Severity Index; PSSD, Psoriasis Symptoms and Signs Diary; SD, standard deviation; sPGA, static Physician Global Assessment.

^a^ One patient in the population with GPP had an sPGA score of 1; the inclusion criteria for this cohort did not require sPGA scores ≥3.

### 3.2 PSSD

#### 3.2.1 PP

**FIGURE 1:**
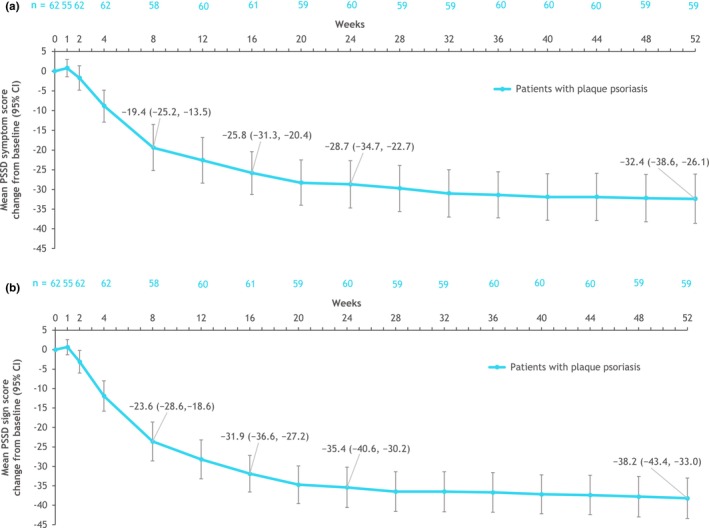
PSSD symptom (a) and sign (b) score changes from baseline^a^ in patients with plaque psoriasis over 52 weeks (as observed). ^a^Mean (SD) baseline PSSD symptom score: 39.8 (23.6); mean (SD) baseline PSSD sign score: 48.0 (19.5). CI, confidence interval; PSSD, Psoriasis Symptoms and Signs Diary; SD, standard deviation.

From mean baseline PSSD symptom and sign scores of 39.8 (SD, 23.6) and 48.0 (SD, 19.5), respectively, in the PP group, the mean (95% confidence interval [CI]) symptom and sign score changes at week 16 were − 25.8 (95% CI, −31.3 to −20.4) and − 31.9 (95% CI, −36.6 to −27.2), respectively (Figure 1). Further numeric improvement was reported at week 52 in each summary score (symptom: −32.4 [95% CI, −38.6 to −26.1]; sign: −38.2 [95% CI, −43.4 to −33.0]).

At week 16, the response rate for meaningful change on PSSD individual items ranged from 31.7% (95% CI, 20.6–44.7) for the symptom item stinging to 76.2% (95% CI, 63.8–86.0) for the sign item shedding or flaking. At week 52, response rates for all individual items had numerically increased from their week 16 levels; 73.0% of patients in the PP group reported meaningful change in the symptom itch, while 87.3% reported meaningful improvement in skin shedding or flaking.

#### 3.2.2 GPP

From mean baseline PSSD symptom and sign scores of 46.6 (SD, 4.0) and 57.3 (SD, 20.3), respectively, mean symptom and sign score changes from baseline at week 16 were − 23.4 (95% CI, −42.8 to −4.0) and − 25.8 (95% CI, −44.8 to −6.8), respectively, in the three patients with GPP. Mean symptom and sign score changes from baseline were − 16.4 (95% CI, −76.3 to 43.5) and − 22.9 (95% CI, −98.5 to 52.8), respectively, in the two patients with recorded scores at week 52.

#### 3.2.3 EP

From mean baseline PSSD symptom and sign scores of 35.7 (SD, 26.0) and 47.1 (SD, 19.0), respectively, mean symptom and sign score changes from baseline at week 16 were −22.0 (95% CI, −49.6 to 5.6) and −26.9 (95% CI, −51.3 to −2.5), respectively, in the seven patients with EP with observable data. At week 52, the six observable patients reported mean symptom and sign score changes from baseline of −27.3 (95% CI, −64.2 to 9.6) and −31.4 (95% CI, −61.6 to −1.3), respectively.

### 3.3 DLQI

From mean baseline scores of 9.1 (SD, 4.5), 9.7 (SD, 6.4), and 7.5 (SD, 5.6) in the PP, GPP, and EP groups, respectively, mean DLQI score change at week 16 ranged from −6.6 to −8.0. In each group, these improvements were numerically increased by the end of the trial: week 52 mean DLQI score changes from baseline were −7.6 (95% CI, −8.8 to −6.4), −8.3 (95% CI, −27.0 to 10.3), and −7.8 (95% CI, −13.8 to −1.9) in the PP, GPP, and EP groups, respectively.

At week 16, the response rates for ≥4‐point meaningful change from baseline in DLQI scores were 77.4% (95% CI, 65.0–87.1), 100.0% (95% CI, 29.2–100.0), and 57.1% (95% CI, 18.4–90.1) in the PP, GPP, and EP groups, respectively. At week 52, these rates were maintained or numerically increased in each group (83.3% [95% CI, 71.5–91.7], 100.0% [95% CI, 29.2–100.0], and 66.7% [95% CI, 22.3–95.7] in the PP, GPP, and EP groups, respectively).

**FIGURE 2:**
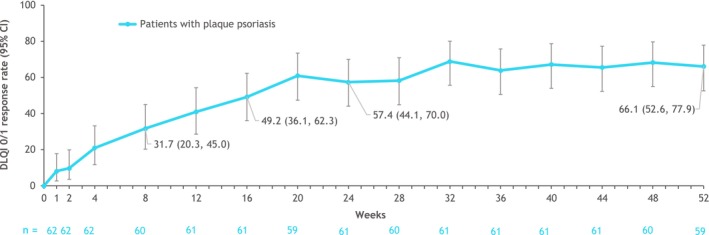
DLQI 0/1 response rates^a^ in patients with plaque psoriasis over 52 weeks (as observed). ^a^Proportion of patients with a baseline DLQI score of ≥2 who reported DLQI 0/1. CI, confidence interval; DLQI 0/1, Dermatology Life Quality Index scores of 0 or 1.

The DLQI 0/1 response rate in the PP group was 49.2% (95% CI, 36.1–62.3) at week 16 and 66.1% (95% CI, 52.6–77.9) at week 52 (Figure 2). The week 52 response rate was similar across the three psoriasis groups (66.1%–66.7%), although with wide CIs associated with the smaller GPP and EP sample sizes.

## 4 DISCUSSION

Patients in all three psoriasis groups reported improvements in PSSD symptom and sign summary scores. Qualitative research in psoriasis has shown that symptoms and signs of psoriasis contribute to patients' poor QoL; itch has been reported to be the most bothersome symptom of the disease, while skin shedding or flaking is associated with embarrassment, shame, and a sense of stigmatization.^12^, ^13^ At week 52 in POETYK PSO‐4, 73.0% of patients with PP experienced meaningful improvement in itch, while 87.3% reported meaningful improvement in skin shedding and flaking. At week 52, approximately two‐thirds of patients in each group reported no impact of disease on their QoL.

Improvements in patient‐reported outcomes were observed in patients across all three phenotypes of psoriasis; however, the small sample size of patients with GPP or EP warrants cautious interpretation of the results in these patients. Further limitations include the lack of a control group and the open‐label study design.

## 5 CONCLUSION

Japanese patients with PP, GPP, or EP treated with deucravacitinib reported improvements in their psoriasis symptoms and signs, as well as in QoL, over 52 weeks. These findings support the overall clinical efficacy profile of deucravacitinib in Japanese patients with each of these psoriasis phenotypes.

## CONFLICT OF INTEREST STATEMENT

Y.T. has received research grants from AbbVie, Amgen, Boehringer Ingelheim, Bristol Myers Squibb, Eisai, Jimro, Kyowa Kirin, Leo Pharma, Lilly, Maruho, Sun Pharma, Taiho Pharmaceutical, Tanabe‐Mitsubishi, Torii Pharmaceutical, and UCB; honoraria from AbbVie, Amgen, Boehringer Ingelheim, Bristol Myers Squibb, Eisai, Janssen, Jimro, Lilly, Kyowa Kirin, Leo Pharma, Maruho, Novartis, Pfizer, Sun Pharma, Taiho Pharmaceutical, Tanabe‐Mitsubishi, Torii Pharmaceutical, and UCB; and consulting fees from AbbVie, Boehringer Ingelheim, Bristol Myers Squibb, Janssen, Lilly, Maruho, Novartis, Taiho Pharmaceutical, and UCB. A.W.A. has served as a research investigator, scientific advisor, and/or speaker for AbbVie, Almirall, Arcutis, Aslan Pharmaceuticals, Beiersdorf, Boehringer Ingelheim, Bristol Myers Squibb, Dermavant, Dermira, EPI Health, Incyte, Janssen, Leo Pharma, Lilly, Mindera Health, Nimbus, Novartis, Ortho Dermatologics, Pfizer, Regeneron, Sanofi, Sun Pharma, and UCB. S.I. has received grants and personal fees from AbbVie, Eisai, Janssen, Kyowa Kirin, Leo Pharma, Maruho, Sun Pharma, Taiho Yakuhin, Tanabe Mitsubishi, and Torii Yakuhin; and personal fees from Amgen (Celgene), Boehringer Ingelheim, Bristol Myers Squibb, Daiichi Sankyo, GSK, Lilly, Novartis, and UCB. Y.O. has received research grants from AbbVie, Eisai, Maruho, Shiseido, Sun Pharma, and Torii; received honoraria from AbbVie, Amgen, Boehringer Ingelheim, Bristol Myers Squibb, Eisai, Janssen Pharma, Jimro, Kyowa Kirin, Leo Pharma, Lilly, Maruho, Novartis Pharma, Pfizer, Sanofi, Sun Pharma, Taiho Pharmaceutical, Tanabe‐Mitsubishi, Torii Pharmaceutical, and UCB; and participated as an investigator in clinical trials for AbbVie, Amgen, Boehringer Ingelheim, Bristol Myers Squibb, Janssen Pharma, Leo Pharma, Lilly, Maruho, Pfizer, Sun Pharma, and UCB. A.M. has received honoraria as a meeting chair/lecturer for AbbVie, Boehringer Ingelheim Japan, Eisai, Eli Lilly Japan K.K., Inforward, Janssen Pharmaceutical K.K., Kyowa Kirin, Maruho Co., Nippon Kayaku, Novartis Pharma K.K., Taiho Pharmaceutical, Torii Pharmaceutical, and Ushio; funding/grants from AbbVie G.K., Eisai, Eli Lilly Japan K.K., Kyowa Hakko Kirin, Leo Pharma K.K., Maruho, Mitsubishi Tanabe Pharma, Novartis Pharma K.K., Taiho Pharmaceutical, and Torii Pharmaceutical; and consulting fees from AbbVie GK, Boehringer Ingelheim Japan, Bristol Myers Squibb, Celgene K.K., Eli Lilly Japan K.K., Janssen Pharmaceutical K.K., Kyowa Kirin, Maruho, Nichi‐Iko Pharmaceutical, Nippon Kayaku, Novartis Pharma K.K., Pfizer Japan, Torii Pharmaceutical, and UCB Japan. Y.Z., J.Z., B.B., and A.N. are employees of and shareholders in Bristol Myers Squibb. S.B. was an employee of and shareholder in Bristol Myers Squibb at the time of the study. M.O. has received honoraria and/or research grants from AbbVie, Amgen, Boehringer Ingelheim, Bristol Myers Squibb, Eisai, Janssen, Kyowa Kirin, Leo Pharma, Lilly, Maruho, Mitsubishi Tanabe Pharma, Nichi‐Iko, Nippon Kayaku, Novartis, Pfizer, Sanofi, Sun Pharma, Taiho Pharmaceutical, Torii Pharmaceutical, and UCB. Y.T and S.I. are Editorial Board members of the *Journal of Dermatology* and co‐authors of this article. To minimize bias, they were excluded from all editorial decision‐making related to the acceptance of this article for publication.

## DATA SHARING STATEMENT

The data sets generated during and/or analyzed during the current study are not publicly available, but requests for the data will be considered. Please see the Bristol Myers Squibb policy on data sharing for more information: https://www.bms.com/researchers‐and‐partners/independent‐research/data‐sharing‐request‐process.html.
